# Is There a Relationship Between Unilateral/Bilateral Impacted Maxillary Canines and Nasal Septum Deviation?

**DOI:** 10.7759/cureus.47931

**Published:** 2023-10-29

**Authors:** Ebru Kucukkaraca

**Affiliations:** 1 Department of Orthodontics, Faculty of Dentistry, Ankara Yildirim Beyazit University, Ankara, TUR

**Keywords:** cbct imaging, maxillary width, unilateral impacted tooth, impacted maxillary canine, nasal septal deviation

## Abstract

Introduction

A deviated nasal septum may be associated with some dentofacial deformities. The aim of the study was to determine whether there is a relationship between some craniomaxillary features of unilateral and bilateral maxillary impacted canines and nasal septum deviation.

Methods

This is a retrospective study consisting of cone beam computed tomography (CBCT) images of 51 patients. All patients were divided into three subgroups: unilateral maxillary impacted canines (UMIC) (n=19) bilateral maxillary impacted canines (BMIC) (n=15), and control group (MC) (n=17). The septal deviation angle and some angular and dimensional measurements were performed. Differences in linear and angular measurements between the groups were analyzed using One-way ANOVA and the Kruskal-Wallis test. Pearson's correlation analysis was performed to determine the relationship between the septal deviation angle, septal deviation direction, nasal floor angle, and other parameters, and multivariate linear regression analysis was performed to determine the effect of variables in the septal deviation angle.

Results

Bilateral or unilateral position of the impacted canines was found to be effective on septal deviation. The septal deviation angle and the nasal floor angle values were found to be significantly higher in the UMIC and BMIC groups (p<0.001) than in the MC group. Maxillary width was found to be significantly lower in the BMIC group compared to the UMIC (p<0.01) and MC group (p<0.001). Septal deviation angle was positively correlated with septal deviation direction and nasal floor angle (p<0.001). Palatal width and nasal floor angle were found to be negatively correlated (p<0.05), and palatal depth and septal deviation direction were found to be positively correlated (p<0.01). Groups and septal deviation angle, septal deviation direction, and nasal floor angle were found to be negatively correlated (p<0.001). The multivariate linear regression analysis revealed an association between septal deviation angle, group (p<0.01), and nasal floor angle (p<0.05).

Conclusion

Bilateral or unilateral position of the impacted canines was found to be effective on septal deviation. The septal deviation angle values were found to be higher when the maxillary impacted canine was unilateral. Unilateral or bilateral positions of the impacted canine and the nasal floor angle were found to be factors affecting the formation of septal deviation.

## Introduction

The relationship between the development of craniofacial structures and respiratory function has been a topic of interest and discussion in recent years. When nasal airway obstruction occurs due to various reasons, a compulsory mouth-breathing habit occurs [1]. The most common obstructive causes of this are hypertrophic adenoids, maxillary sinusitis, concha bullosa (CB), deviated nasal septum, and hypertrophic inferior turbinate [2]. Gray [3] reported that the incidence of deviated septum was 58% in infants and 79% in adults in a study of 2,380 infants and 2,112 adults.

There have been important anatomical connections between the maxilla and nasal structures since the initial stages of growth and development. Septal deviations may occur due to prenatal, postnatal, and childhood traumas, asymmetrical development as a result of abnormal pressures related to the position of the fetus, and problems in the development of the surrounding bone tissue caused by any factor [4,5].

A deviated nasal septum causes a restriction of nasal airflow. Restricted nasal airflow leads to mouth breathing. Mouth breathing causes some incompatible developments in the craniofacial structures. As a result of incompatible development that occurs with oral breathing, facial shapes called “adenoid face” or “long face syndrome” may occur. Studies on this subject have pointed out the role of enlarged adenoids and tonsils and allergic rhinitis on the breathing pattern [6]. In children who underwent adenoidectomy/tonsillectomy, it has been reported that at the end of five years of follow-up, there were improvements in the mandibular plane angle, maxillary width, and incisor tooth positions, and breathing returned to normal [7].

Chronic mouth breathing results in a narrowing of the upper jaw due to negative air pressure. This eventually leads to the appearance of some dentofacial deformities and malocclusions [8]. Due to the narrowing of the upper jaw and the decrease in arch size required for tooth eruption, canine tooth eruption may be affected and may remain ectopic or impacted [9-12]. The study by Schindel and Duffy reported that the rate of ectopic and impacted canines was higher in patients with transversal incompatibility and that this was generally unilateral [13]. D'ascanio et al. [14] analyzed children with nasal septal deviation and showed that facial and palatal height increased and maxillary intermolar width was narrowed compared to the control group. The narrowness of the maxillary arch can cause crowding in the transition to permanent dentition and can cause the permanent maxillary canines, which erupt at around 11-13 years of age, not to erupt in the appropriate position in the arch and to remain impacted [15,16].

Ballanti et al. reported that there was no relationship between maxillary transversal deficiency and nasal septum deviation in prepubertal individuals [4]. Determining the presence or absence of nasal septal deviation and impacted canine relationship will benefit the clinician in terms of many orthodontic, aesthetic, and occlusion-related factors. The growth and development of the nasomaxillary complex and the development of the teeth cannot be separated. Any problem in either development will definitely affect the other. The aim of the study was to determine whether there is any relationship between some craniomaxillary features of unilateral and bilateral maxillary impacted canines and nasal septum deviation. The null hypothesis of the study was established as “there is no relationship between maxillary impacted canines and nasal septal deviation angle.”

## Materials and methods

Ethics approval

The research was approved by the ethics committee of Ankara Yildirim Beyazit University, Ankara, Turkey (ref: 13.04.2023/04).

Sample size calculation and participants

The sample size was calculated using the G*power software (version 3.1.9; Franz Faul Universitat, Kiel, Germany). Power analysis before the study was calculated with reference to the intercanine width, as evaluated in the study by Sharhan et al. [9]. According to the power analysis, the total sample size was determined to be 30, with a desired power (1-b) of 0.95 at the conventional a level (0.05) and an effect size of 0.77. According to the post hoc power analysis performed after the study, the total sample size was determined to be 48, with a desired power (1-b) of 0.95 at the conventional a level (0.05) and an effect size of 2.81.

The study groups consisted of cone beam computed tomography (CBCT) images obtained from a total of 51 patients who applied for treatment of impacted teeth at Ankara Yıldırım Beyazıt University, Faculty of Dentistry, Department of Orthodontics, and who met the inclusion criteria.

Study design and eligibility criteria of participant

Inclusion/Exclusion Criteria

Adolescents with unilateral/bilateral maxillary impacted canines (aged 9-16) and CBCT images with good image quality were included in the study, while individuals with any syndrome in the craniofacial region or an anomaly affecting the craniofacial region, pathological lesions in the craniofacial region, multiple missing teeth, previous orthodontic treatment, and CBCT images with poor image quality were excluded from the study.

Study Groups

Unilateral maxillary impacted canines (UMIC): (n=19), Bilaterally maxillary impacted canines (BMIC) (the impacted canines on both sides are positioned in the same position, either buccal or palatinal): (n=15), Control (MC) (consisted of properly erupted maxillary canines that met the inclusion criteria and were not impacted): (n=17).

Definitions of the variables and measurement procedure

**Figure 1:**
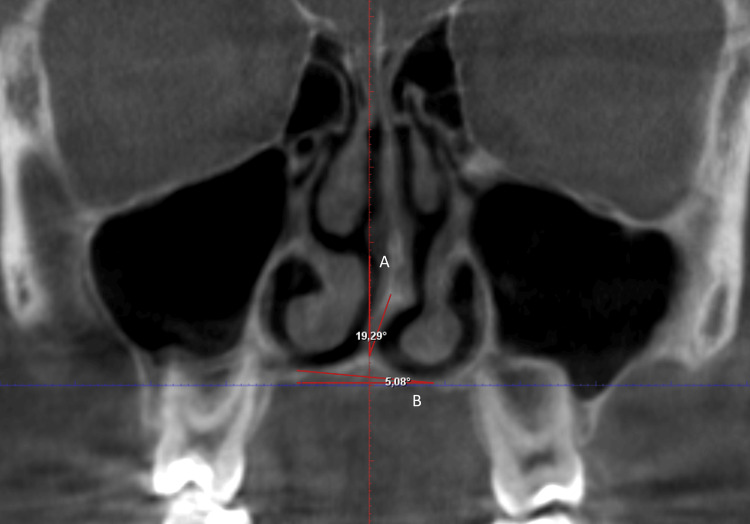
A. The septal deviation angle (SDA) is the angle between the line drawn from the crista galli to the ANS and the line drawn from the crista galli to the point of maximum deviation on the septum in coronal section. B. Nasal floor angle (NFA) is the angle between the horizontal plane passing through the ANS and the plane tangent to the floor of the nasal septum in coronal section.

**Figure 2:**
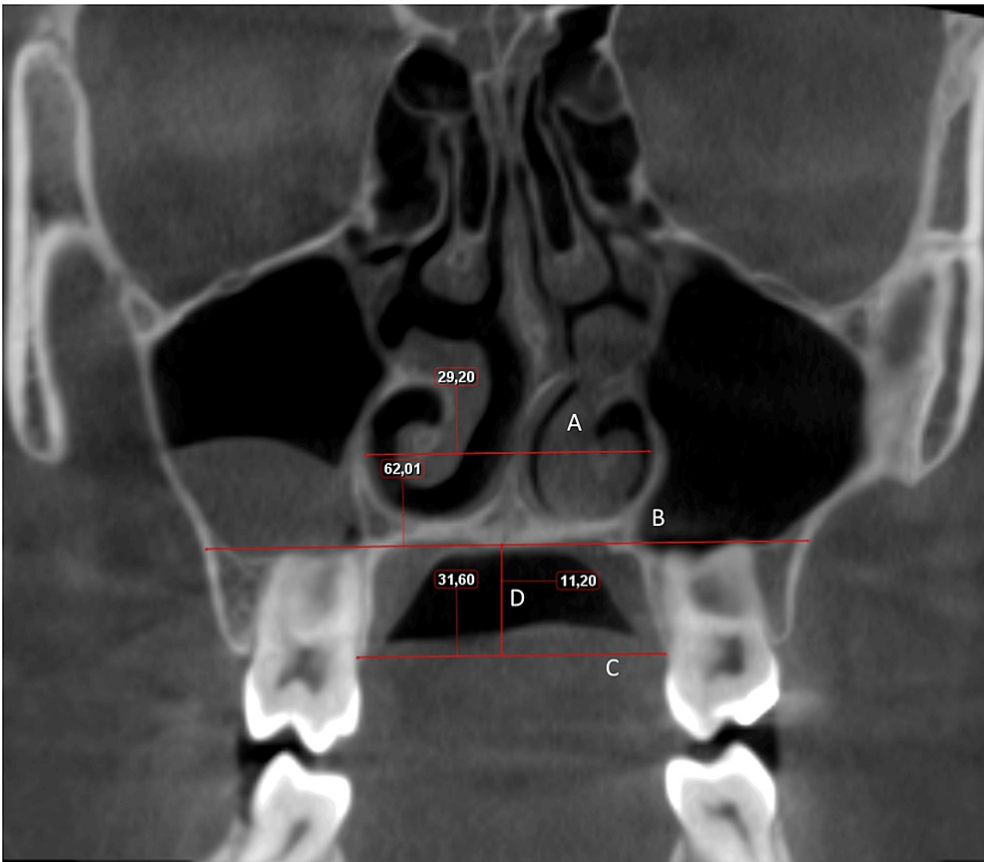
A. Nasal floor width (NFW): The width between the widest points of the floor of the nasal septum in coronal section. B. Maxillary width (MW): Horizontal distance between the right and left maxillary deepest points in coronal section. C. Palatal width (PW): horizontal distance between the cemento enamel junction (CEJ) of the right and left maxillary 1st molars in coronal section. D. Palatal depth (PD): vertical distance from the midpalatal suture to the horizontal plane between the cemento enamel junction (CEJ) of the right and left maxillary 1st molars in coronal section.

**Figure 3:**
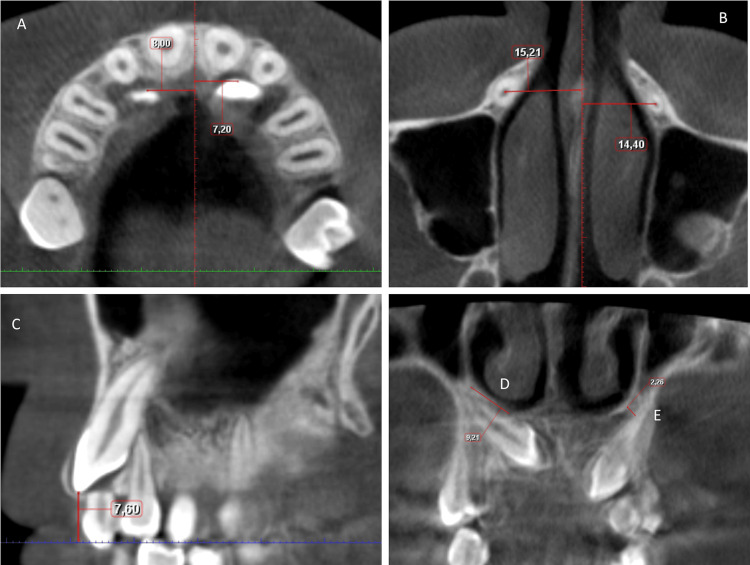
A. U3cusp-midline distance (U3CMD): the horizontal distance from the maxillary canine crown tip to the midline in axial section. B. U3root-midline distance (U3RMD): the horizontal distance from the maxillary canine root tip to the midline in axial section. C. U3-occ. distance (U3OD): vertical distance from the maxillary canine crown tip to the occlusal plane in sagittal section. D. U3root-nasal cont. surface (U3NC): The amount of contact surface of the maxillary canine root with the nasal cavity in coronal section. E. U3root-nasal cavity distance (U3ND): The closest distance of the maxillary canine root to the nasal cavity in coronal section.

The variables and definitions used in the study are given in Table 1. All angular and dimensional measurements were performed in Planmeca Romexis 3D Imaging Software (Planmeca Romexis 3D Imaging Software, Helsinki, Finland) (Figures 1-3).

**Table 1:** Definitions of the variables used in the study

Variable	Definition
The septal deviation angle (SDA) (Figure 1.A)	the angle between the line drawn from the crista galli to the ANS and the line drawn from the crista galli to the point of maximum deviation on the septum in the coronal section.
Nasal floor angle (NFA) (Figure 1.B)	the angle between the horizontal plane passing through the ANS and the plane tangent to the floor of the nasal septum in the coronal section.
Nasal floor width (NFW) (Figure 2.A)	the width between the widest points of the floor of the nasal septum in the coronal section.
Maxillary width (MW) (Figure 2.B)	horizontal distance between the right and left maxillary deepest points in the coronal section.
Palatal width (PW) (Figure 2.C)	horizontal distance between the cemento enamel junction (CEJ) of the right and left maxillary 1st molars in the coronal section.
Palatal depth (PD) (Figure 2.D)	vertical distance from the midpalatal suture to the horizontal plane between the cemento enamel junction (CEJ) of the right and left maxillary 1st molars in the coronal section.
U3-U3 arch width (U3-U3W) (Figure 3.A)	distance between the right and left maxillary canine crown tips in the axial section.
U6-U6 arch width (U6-U6W) (Figure 3.B)	distance between the right and left maxillary 1st molar sulcus in the axial section.
Maxillary arch length (AL) (Figure 3.C)	distance from the incisal foramina to the line that connects the sulcus midpoints of the right and left first molars in the axial section.
U3cusp-midline distance (U3CMD) (Figure 4.A)	the horizontal distance from the maxillary canine crown tip to the midline in the axial section.
U3root-midline distance (U3RMD) (Figure 4.B)	the horizontal distance from the maxillary canine root tip to the midline in the axial section.
U3-occ. distance (U3OD)(Figure 4.C)	vertical distance from the maxillary canine crown tip to the occlusal plane in the sagittal section.
U3root-nasal cont. surface (U3NC) (Figure 4.D)	the amount of contact surface of the maxillary canine root with the nasal cavity in the coronal section.
U3root-nasal cavity distance (U3ND) (Figure 4.E)	the closest distance of the maxillary canine root to the nasal cavity in the coronal section.

Statistical analysis

The data collected were analyzed using IBM SPSS Statistics for Windows, Version 24.0 (IBM Corp., Armonk, NY, USA). The demographic data were analyzed using the chi-square test. The normality distribution analysis of the measurements was performed using the Shapiro-Wilk test. Differences in linear and angular measurements between the groups were analyzed using One-way ANOVA for the values that fit the normal distribution and Kruskal-Wallis test for the values that did not fit the normal distribution. Post-hoc tests were used for homogeneous ones; Tukey test and Tamhane's T2 test were used for those not homogeneously distributed. Pearson's correlation analysis was performed to determine the relationship between the septal deviation angle, nasal floor angle, and other parameters. The multivariate linear regression analysis was performed using regression analysis. P<0.05 was considered to be significant.

## Results

In the UMIC group (n=19), 10 females and nine males had a mean age of 15.21±0.53 years. In the BMIC group (n=15), eight females and seven males had a mean age of 15.06±0.36 years. In the control group (n=17), nine females and eight males had a mean age of 15.14±0.41 years. The septal deviation was 27.5% (n=14) on the right side, 31.4% (n=16) on the left side, and 41.2% (n=21) non-deviation. The canines were buccally placed in 17 (33.3%), palatally placed in 17 (33.3%), and normally placed in 17 (33.3%) of the participants with impacted canines. A statistically significant positive association was found between the groups and the U3 impaction side (X^2^=51.179, p<0.001) and septal deviation direction (X^2^=18.873, p<0.01) (Table 2).

**Table 2:** The results of the demographic statistics according to groups ^a^ Pearson’s chi-square, ^b ^Fisher’s exact test, df: degrees of freedom.

	Unilateral (UC) n:19	Bilateral (BC) n:15	Control (CC) n:17	Total N: 51	X^2^	df	p
Age Mean±SD	15.21±0.53	15.06±0.36	15.17±0.35	15.14±0.41	17.47^a^	16	p>0.05
Gender n (%)					0.002^a^	2	p>0.05
Female	10 (52.6%)	8 (53.3%)	9 (52.9%)	27 (52.9%)			
Male	9 (47.4%)	7 (46.7%)	8 (47.1%)	24 (47.1%)			
U3 Impaction side n (%)					51.179^a^	4	p<0.001
buccal side	9 (47.4%)	8 (53.3%)	0 (0%)	17 (33.3%)			
palatinal side	10 (52.6%)	7 (46.7%)	0 (0%)	17 (33.3%)			
normal	0 (0%)	0 (0%)	17 (33.3%)	17 (33.3%)			
Septal deviation direction					18.873^b^	-	p<0.001
Rigth n (%)	6 (31.6%)	6 (40%)	2 (11.8%)	14 (27.5%)			
Left n (%)	10 (52.6%)	5 (33.3%)	1 (5.9%)	16 (31.4%)			
Non-deviation n (%)	3 (15.8%)	4 (26.7%)	14 (82.4%)	21 (41.2%)			

The value of the septal deviation angle was found to be significantly greater in the UMIC (8.82º ±1.25) (p<0.001) and BMIC groups (6.34 º ±1.01) (p<0.01) than in the MC group (2.34 º ±0.45). The nasal floor angle was found to be significantly greater in the UMIC and BMIC groups than in the MC group (p<0.001). The septal deviation angle was positively correlated with the direction of septal deviation and nasal floor angle (p<0.001) (Tables 3, 4).

**Table 3:** The mean values and standard deviation of measurements and comparison between groups ^a^One-way ANOVA test for normally distribution. ^b^Kruskal-Wallis test for non-normally distribution. Post-hoc for homogeneous ones: Tukey test. +Tamhane's T2 Test for those not homogeneously distributed. p<0.05 statistically significant Nasal Floor Width (NFW), Maxillary Width (MW), Palatal Width (PW), Palatal Depth (PD), Septum Deviation Angle (SDA), Nasal Floor Angle (NFA), U3-Nasal Cavity Distance (U3ND), U3-Nasal Contact Surface (U3NC), U3-U3 Arch Width (U3-U3W), U6-U6 Arch Width (U6-U6W), Maxillary Arch Length (AL), U3-Occ. Distance (U3OD), U3Cusp-Midline Distance (U3CMD), U3Root-Midline Distance (U3RMD).

	Unilateral (UMIC)	Bilateral (BMIC)	Control (MC)		Post Hoc		
	Mean±SD	Mean±SD	Mean±SD	p	UMIC-BMIC	UMIC-MC	BMIC-MC
Septal dev. angle	8.82±1.25	6.34±1.01	2.34±0.45	<0.001^b^	0.355	<0.001	0.006
Nasal floor angle	3.71±0.42	3.66±0.40	1.56±0.27	<0.001^b^	0.990	<0.001	<0.001
Nasal floor width	31.04±0.73	29.87±0.90	31.32±0.75	0.425^a^	0.560	0.964	0.429
Maxillary width	62.23±0.52	59.48±0.60	62.65±0.55	<0.001^a^	0.003	0.847	<0.001
Palatal width	34.90± 0.72	32.98±0.72	36.01±0.40	0.008^a^	0.099	0.430	0.006
Palatal depth	12.48±0.40	11.57±0.48	10.66±0.26	0.006^a^	0.244	0.004	0.267
U3-nasal cavity dis.	1.30±0.53	1.01±0.30	7.47±0.36	<0.001^b^	0.956	<0.001	<0.001
U3-nasal cont. surf.	1.55±0.32	1.71±0.35	0000±0.00	<0.001^a^	0.906	<0.001	<0.001
U3-U3 arch width	33.40±0.68	30.16±0.53	35.16±0.35	<0.001^a^	<0.001	0.067	<0.001
U6-U6 arch width	46.40±0.57	44.76±0.77	46.90±0.53	0.059^a^	0.165	0.827	0.058
Maxillary arch length	27.55±0.56	26.57±0.55	28.42±0.45	0.066^b^	0.530	0.552	0.051
U3-occ. distance	9.50±0.82	6.61±0.66	0000±0.00	<0.001^a^	0.007	<0.001	<0.001
U3cusp-midline dis.	10.13±1.24	11.52±0.93	8.11±0.46	0.034^b^	0.761	0.371	0.011
U3root-midline dis.	14.78±0.45	14.05±0.63	12.50±0.58	0.006^b^	0.720	0.013	0.229

**Table 4:** Correlations between septal deviation angle, septal deviation side and nasal floor angle and parameters Data were analyzed using Pearson’s correlation analysis. Nasal Floor Width (NFW), Maxillary Width (MW), Palatal Width (PW), Palatal Depth (PD), Septum Deviation Angle (SDA), Nasal Floor Angle (NFA), U3-Nasal Cavity Distance (U3ND), U3-Nasal Contact Surface (U3NC), U3-U3 Arch Width (U3-U3W), U6-U6 Arch Width (U6-U6W), Maxillary Arch Length (AL), U3-Occ. Distance (U3OD), U3Cusp-Midline Distance (U3CMD), U3Root-Midline Distance (U3RMD).

	Septal dev. angle		Septal dev. direction		Nasal floor angle	
	r	p	r	p	r	p
Group	-0.559	<0.001	-0.558	<0.001	-0.493	<0.001
Gender	0.071	0.310	0.156	0.275	0.216	0.064
Septal dev. angle	1.000	.	0.661	<0.001	0.508	<0.001
Nasal floor angle	0.508	<0.001	0.450	0.001	1.000	.
Nasal floor width	0.009	0.476	-0.082	0.567	-0.210	0.069
Maxillary width	-0.101	0.241	-0.207	0.146	-0.196	0.085
Palatal width	-0.100	0.243	-0.176	0.216	-0.292	0.019
Palatal depth	0.124	0.192	0.362	0.009	0.169	0.118
U3-nasal cavity dis.	-0.543	<0.001	-0.439	0.001	-0.483	<0.001
U3-nasal cont. surf.	0.299	0.016	0.329	0.018	0.388	0.002
U3-U3 arch width	-0.213	0.067	-0.321	0.022	-0.139	0.165
U6-U6 arch with	-0.046	0.373	-0.123	0.389	-0.152	0.143
Maxillary arch length	-0.255	0.035	-0.183	0.199	0.104	0.234
U3-occ. distance	0.450	<0.001	0.495	<0.001	0.476	<0.001
U3cusp-midline dis.	0.150	0.147	0.019	0.894	0.299	0.017
U3root-midline dis.	0.267	0.029	0.289	0.040	0.189	0.092
U3 impaction side	0.051	0.723	0.539	< .001>	-0.241	0.088

The maxillary width was found to be significantly smaller in the BMIC group compared to the UMIC (p<0.01) and MC groups (p<0.001). The palatal width value was found to be statistically significantly lower in the BMIC group than in the MC group (p<0.01), while the palatal depth value was found to be significantly higher in the UMIC group than in the MC group (p<0.01). The palatal width and nasal floor angle were found to be negatively correlated (p<0.05), and the palatal depth and the direction of the septal deviation were found to be positively correlated (p<0.01) (Tables 3, 4).

The U3-nasal cavity distance was found to be significantly lower in the UMIC and BMIC groups than in the MC group (p<0.001). Canine roots were placed closer to the nasal cavity in the impacted groups. U3-nasal distance and U3-occ. distance was correlated with septal deviation angle, septal deviation direction, and nasal floor angle (p<0.001) (Tables 3, 4).

U3-nasal cont. surface length was found to be greater in the UMIC and BMIC groups than in the MC group (p<0.001). U3-nasal cont. surface length was correlated with the septal deviation angle, the septal deviation direction, and the nasal floor angle (Tables 3, 4).

U3-U3 arch width (p<0.001) was found to be smaller in the BMIC group compared to the other groups, while no difference was found in the U6-U6 arch width. U3-U3 arch width and septal deviation direction were found to be negatively correlated (p<0.05). U3-occ. distance was significantly greater in the BMIC and UMIC groups (p<0.001), while U3cusp-midline distance was significantly greater in the BMIC group (p<0.05) and U3 root-midline distance was significantly greater in the UMIC group (p<0.05). U3cusp-midline distance and nasal floor angle were found to be positively correlated (p<0.05). U3root-midline distance, septal deviation angle, and septal deviation direction were found to be positively correlated (p<0.05) (Tables 3, 4).

Arch length and septal deviation angle were found to be positively correlated (p<0.05). U3 impaction side and septal deviation direction were found to be positively correlated (p<0.001). The groups and the septal deviation angle, the septal deviation direction, and the nasal floor angle were found to be negatively correlated (p<0.001). Multivariate linear regression analysis revealed an association between septal deviation angle, groups (p<0.01), and nasal floor angle (p<0.05) (Table 5).

**Table 5:** Multivariate Linear Regression analysis of variables with septal deviation angle Nasal Floor Width (NFW), Maxillary Width (MW), Palatal Width (PW), Palatal Depth (PD), Septum Deviation Angle (SDA), Nasal Floor Angle (NFA), U3-Nasal Cavity Distance (U3ND), U3-Nasal Contact Surface (U3NC), U3-U3 Arch Width (U3-U3W), U6-U6 Arch Width (U6-U6W), Maxillary Arch Length (AL), U3-Occ. Distance (U3OD), U3Cusp-Midline Distance (U3CMD), U3Root-Midline Distance (U3RMD).

		Unstandardized Coefficients		Standardized Coefficients	t	Sig.	95.0% Confidence Interval for B		
		B	Std. Error	Beta			Lower Bound	Upper Bound	
	Group	-3.030	1.092	-0.525	-2.776	0.008	-5.227	-0.834	
	Gender	2.461	1.612	0.254	1.527	0.136	-0.812	5.733	
	Nasal floor angle	1.078	0.449	0.402	2.403	0.022	0.167	1.989	
	Nasal floor width	0.174	0.265	0.116	0.657	0.516	-0.364	0.712	
	Maxillary width	0.024	0.324	0.013	0.073	0.942	-0.634	0.682	
	Palatal width	0.137	0.358	0.080	0.384	0.704	-0.589	0.863	
	Palatal depth	-0.501	0.507	-0.181	-0.988	0.330	-1.530	0.528	
	U3-nasal cavity dis.	-0.648	0.352	-0.461	-1.842	0.074	-1.363	0.066	
	U3-nasal cont. surf.	-0.674	0.730	-0.188	-0.923	0.362	-2.157	0.808	
	U3-U3 arch with	0.019	0.336	0.012	0.056	0.956	-0.664	0.701	
	U6-U6 arch with	0.128	0.360	0.070	0.355	0.725	-0.601	0.857	
	Maxillary arch length	-0.760	0.376	-0.350	-2.021	0.051	-1.523	0.004	
	U3-occ. distance	0.260	0.299	0.257	0.870	0.390	-0.347	0.867	
	U3cusp-midline dis.	-0.011	0.184	-0.010	-0.061	0.952	-0.385	0.363	
	U3root-midline dis.	-0.029	0.351	-0.014	-0.082	0.935	-0.741	0.683	
	U3 impaction side	0.268	1.676	0.028	0.160	0.874	-3.133	3.670	

## Discussion

A deviated nasal septum causes a restriction of nasal airflow. Restricted nasal airflow leads to mouth breathing. The relationship between mouth breathing and malocclusion caused by a deviated nasal septum is well known, and studies have attempted to explain its effect on various structures. Many studies have described the relationship between nasal septal deviation and maxillary transversal dimensions, maxillary sinus volumes, and impacted teeth [17-20]. These studies generally emphasized the possibility that septal deviation may lead to some malocclusions. However, the effect of the unilateral or bilateral position of the impacted canines on septal deviation has not been investigated. To complete this deficiency in the literature, this study aimed to investigate the relationship between unilateral or bilateral maxillary impacted canines and septal deviation.

Malocclusions that can occur due to the nasal septum deviation are increased palatal depth and overjet, decreased maxillary and intermolar width, and posterior crossbite [14]. At the same time, nasal septum deviation is a factor affecting nasal bone morphology. In studies, nasal bone length and thickness were found to be greater on the deviation side than on the opposite side, but no correlation was found with the degree of septal deviation [21]. In this study, although the maxillary and palatal widths were smaller in the UMIC and BMIC groups, the septal deviation was found to be related to parameters such as the distance of the canine root to the nasal cavity, the contact surface with the nasal cavity, the distance to the midline (horizontal distance), or the distance to the occlusion plane (vertical distance).

Some studies of CBCT images reported that nasal septal deviation and the presence of impacted teeth were not related to maxillary sinus volume [17]. Other studies reported that the volume of the maxillary sinus was smaller on the same side as the deviation in cases of moderate and severe septal deviation and that the volume of the maxillary sinus tended to decrease with increasing age [18]. In this study, it was found that there was a relationship between unilateral or bilateral positioning of the impacted canine and the septal deviation angle. There is a significant relationship between the septal deviation angle and the unilateral maxillary impacted canine group. Therefore, the null hypothesis of this study is rejected.

Sapmaz et al. [22] found a positive correlation between the septal deviation angle and the palatal angle. In this study, the septal deviation angle and the nasal floor angle were positively correlated, similar to the study by Sapmaz et al. In some studies in the literature, a relationship was found between nasal septum deviation and buccal displaced canines [23]. Madero et al. [24] reported that bilaterally and unilaterally impacted canine patients had lower transversal width values at the dental level, but they did not find any relationship between maxillary arch width and buccal-palatal position of the impacted canine. In the present study, transversal dimensions (maxillary and palatal width) were statistically significantly smaller in the BMIC group than in the MC group. In addition, the present study found that the septal deviation angle was associated with variables related to the impacted canine rather than the transversal dimensions.

The root apices of palatally impacted canines are positioned more palatally than the root apices of normally erupted canines. There was no difference between unilateral palatally impacted canines and bilateral palatally impacted canines [25]. Yu et al. [26] reported that the mean distance to the midline of palatal-positioned impacted canines was between 5.4 and 8.4 mm. This study did not differentiate between palatally and buccally impacted canines; however, there was no difference in the position of the root tips between the unilateral and bilateral impacted canine groups, but it was found that the root tips were further from the midline in the unilateral impacted canine group (14.78 mm) than in the control group (12.50 mm). Furthermore, a positive correlation was found between the U3root midline distance and the septum deviation angle.

Insufficient arch length and arch length discrepancy may be an early risk factor for buccal canine impaction. In addition, insufficient intermolar width increased palatal depth, and decreased total mesiodistal width of the maxillary incisors were also risk factors of canines for the remaining palatally impacted [27]. One study also suggests that one of the main reasons for impacted maxillary canines is the distance of the cusp tip from the occlusal plane [28]. Kahraman et al. [29] reported that the mean value of the distance of the crown tip of the right and left impacted canines to the occlusal plane was 10 mm higher and the mean distance to the midline was 9 mm. In this study, the distance to the occlusal plane was found to be 9.50 mm in the unilateral impacted canine group and 6.61 mm in the bilateral canine group. The distance from the canine crown to the midline was found to be 10.13 mm in the unilateral group and 11.52 mm in the bilateral group.

Surprisingly, the results of this study show that although the decrease in maxillary transversal dimensions with the effect of mouth breathing and negative air pressure that may occur as a result of septal deviation is a predictive factor for canine impaction, no relationship was found between septal deviation and maxillary transversal width variables. This is similar to the study by Ballanti et al. [4] who found no correlation between septal deviation and maxillary width.

One of the strongest aspects of the present study is that the septal deviation angle was found to be higher in the unilaterally impacted canine compared to the bilaterally impacted canine or control group. This is because both correlation analyses showed a relationship between the amount of nasal contact surface of the impacted canine and the nasal septal deviation. Additionally, according to the results of the regression analysis, the septal deviation angle is affected by the unilateral or bilateral position of the impacted canine and the nasal floor angle. In the literature, there are not many studies investigating the relationship between unilateral or bilateral positioning of the impacted canine and septal deviation. In this respect, this study completes the deficiency in the literature.

Limitations of the study

One of the limitations of this study is that it was performed with 2D measurements. The addition of 3D volumetric measurements, the number of samples, and the separation of the groups into buccal and palatinal impacted canine groups, in addition to unilateral bilateral groups, will contribute to understanding the factors causing septal deviation. In addition, since it was a retrospective study, nasal functions could not be included, and the medical history of the patients who underwent CBCT could not be known completely.

In future studies, more detailed investigations are needed to differentiate whether nasal septal deviation is a predictor of impacted canine formation or whether the presence of impacted canines can lead to nasal septal deviation. More recently, the use of artificial intelligence and machine learning methods may allow automatic segmentation of the maxillary and nasal regions in CBCT images, volumetric measurements, detection of deviations from normal, and access to more detailed information about their causes in future studies [30].

## Conclusions

The bilateral or unilateral positioning of the impacted canines has an effect on the septal deviation. Septal deviation angle values were found to be higher when the maxillary impacted canine was unilateral. The septal deviation angle was not found to be related to the transversal widths of the maxilla. The relationship with septal deviation is more related to the vertical and horizontal positions of the maxillary impacted canine. The unilateral or bilateral position and nasal floor angle of the impacted canine may be the factors in the occurrence of septal deviation.
